# ERdj5 in Innate Immune Cells Is a Crucial Factor for the Mucosal Adjuvanticity of Cholera Toxin

**DOI:** 10.3389/fimmu.2019.01249

**Published:** 2019-06-04

**Authors:** Mee-Sun Kim, Eun-Je Yi, Young-In Kim, So Hee Kim, Yi-Sook Jung, Seong-Ryeol Kim, Takao Iwawaki, Hyun-Jeong Ko, Sun-Young Chang

**Affiliations:** ^1^College of Pharmacy, and Research Institute of Pharmaceutical Science and Technology (RIPST), Ajou University, Suwon-si, South Korea; ^2^Laboratory of Microbiology and Immunology, College of Pharmacy, Kangwon National University, Chuncheon-si, South Korea; ^3^Division of Cell Medicine, Department of Life Science, Medical Research Institute, Kanazawa Medical University, Uchinada, Japan

**Keywords:** cholera toxin (CT), ERdj5, mucosal adjuvant, ER stress, dendrtic cells

## Abstract

Cholera toxin (CT) is one of most strong mucosal adjuvants, but it cannot be clinically used owing to its toxicity. The cytosolic A1 subunit of CT (CTA1) is the molecule responsible for its immunostimulatory activity, which increases the concentration of cyclic AMP and causes the induction of pro-inflammatory cytokines in innate immune cells. However, the importance of endoplasmic reticulum (ER) molecules involved in CTA1 retro-translocation to induce immune responses remained to be investigated. ERdj5 is an ER protein which is expected to transfer CTA1 to the Hrd1 complex for the retro-translocation of CTA1. In this study, we investigated the physiological relevance of ERdj5 in immune stimulation by CT. ERdj5-knockout (ERdj5 KO) mice had decreased production of antigen-specific IgG in the serum and IgA in the mucosal secretion after intranasal immunization with Ag and CT. Especially, IgG2c isotypes were specifically reduced in the absence of ERdj5. ERdj5 KO dendritic cells (DCs) failed to full activation with decreased expression of costimulatory molecules, such as MHC class II, CD80, and CD 86. In ERdj5 KO DCs, secretion of pro-inflammatory cytokines, such as IL-1β, TNF-α, and IL-6, was reduced. The cytokine signatures of several helper T cells were reduced in ERdj5 KO mice following intranasal CT immunization. The absence of ERdj5 affects the immunostimulatory properties of CT but does not affect the response to the CTB pentamer, the response to alum, total antibody production, or cytokine release from DCs exposed to CpG. Interestingly, CT enhanced the expression of ER stress proteins in ERdj5 KO innate immune cells. These results suggested that ERdj5 contributed as a decisive factor to the immunostimulatory capacity of CT via CTA1 retro-translocation.

## Introduction

*Vibrio cholerae* produces an exotoxin (cholera toxin, CT) that causes severe diarrhea in humans (1). CT has been used as a strong mucosal adjuvant when administered with antigen (Ag) via mucosal delivery routes (2, 3). CT is composed of two components, a single A subunit and five B subunit molecules. The B pentamer of CT (CTB) binds to G_M1_ ganglioside on the host cell surface and then enters the host cell. Once bound, the entire CT is endocytosed and the cholera toxin A1 (CTA1) chain is released by the reduction of a disulfide bridge from CTA2 of CTB-CTA2 complex within endoplasmic reticulum (ER). CTA1 is then unfolded, delivered to the Hrd1 complex on ER membrane, and then retro-translocated into the cytoplasm (4). CTA1 binds with a human partner protein called ADP-ribosylation factor 6 (ARF6). ARF6 drives a conformation change of CTA1 which enables its catalytic activity (5). Cytosolic CTA1 can bind to Gsα, catalyzes ADP-ribosylation of Gsα, and finally elevation of 3′, 5′-cyclic AMP (cAMP) concentration in the host cell (6, 7). In the enterocytes, the protein kinase A activated by cAMP phosphorylates the cystic fibrosis transmembrane conductance regulator, a chloride channel protein, leading to ATP-mediated efflux of chloride ions. The ion exchange results in rapid fluid loss from the intestinal tissue to lumen, leading to severe diarrhea.

In the innate immune cells, CT enters the ER of immune cells through endosome following binding with G_M1_ ganglioside, and then the A1 subunit is released via reduction of disulfide bond and CTA1 is retro-translocated to the cytosol. Enhanced cAMP concentration by cytosolic CTA1 induces the production of pro-inflammatory cytokines, including IL-1β, TNF-α, and IL-6 (8–10). In addition, CT induces the expression of costimulatory molecules in Ag presenting cells, such as dendritic cells (DCs) (3, 11). Therefore, the DCs activated by CT induce strong humoral and cellular immunity. Especially, CT can be considered the most powerful mucosal adjuvant because CT can induce strong mucosal IgA responses and T cells (10, 12). The adjuvant effect of CT requires Gsα expression in CD11b^+^ DCs, and promotes a balanced Th1/Th2/Th17 response (3). CT activates DC to produce Th17-driving cytokines, including IL-6, and facilitate the differentiation of Th17 cells (13, 14). However, its strong toxicity limits its use as a practical mucosal vaccine adjuvant (15). To utilize CT as a mucosal vaccine adjuvant candidate with low toxicity, modified CT has been investigated. For instance, CTB itself can be used as an adjuvant without being physically linked to the Ag. Intranasal delivery of CTB combined with ovalbumin (OVA) led to the suppression of Th2 cytokines, enhanced IFN-γ secretion, and OVA-specific IgA production (16). CTB has also been combined with various vaccine Ags, such as the HIV recombinant gp160, to induce protective mucosal IgA responses (17).

ERdj5 is the fifth member of the ERdj family and it contains N-terminal J domain and six tandem thio-redoxin domains (18). ERdj5 as a disulfide reductase mediates redox-assisted regulation of Ca^2+^ homeostasis in the ER and enhances ER-associated degradation (19). ERdj5 has been shown to mediate ER protein quality control in the salivary gland (20). ER molecules involved in protein-folding are induced by ER stress in inflammatory lesions, and they also participate in inflammation and autoimmunity (21). Impaired ERdj5 can be associated with autoimmune manifestation in the salivary gland, such as Sjögren's syndrome (22). ERdj5 is related with protein-folding and translocation across the ER membrane (23). ERdj5 has been shown to be associated with CTA1 retro-translocation (24). ERdj5 is also associated with sel1L in the Hrd-sel1L complex. The binding of ERdj5 to sel1L triggers the interaction of immunoglobulin heavy-chain binding protein (BiP)-toxin to Hrd1 complex, which is proposed to enable CTA1 translocation from the ER to cytosol (24).

CT traffics from the host cell surface to the ER, where catalytic CTA1 subunit retrotranslocates to the cytosol to induce strong toxicity and adjuvanticity. Therefore, the involvement of ERdj5 for CTA1 retro-translocation leads to the question, the role of ERdj5 for immune stimulatory effect by CT. Here, we investigated how ERdj5 deficiency affects mucosal adjuvanticity following intranasal CT administration *in vivo*.

## Materials and Methods

### Mice and Immunization

All experiments were approved by the Institutional Animal Care and Use committee of Ajou University (IACUC approval number 2016-0034). C57BL/6 background mice were purchased from Orient Bio (Sungnam, Korea). ERdj5-knockout (ERdj5 KO) mice were purchased from RIKEN BioResource Center (Ibaraki, Japan) (20). OT-II mice were purchased from Jackson Laboratories (Bar Harbor, ME). The mice were kept during experiment in the Laboratory Animal Research Center of Ajou University Medical Center. The mice were intranasally administered 20 μg OVA and 1 μg CT at a volume of 20 μl at 2 weeks intervals. At 1 week following final immunization, samples including serum, feces, and saliva were collected. Saliva samples were collected by using pipettes with plastic tip after intraperitoneal injection of 5 mg/kg pilocarpine hydrochloride (Sigma-Aldrich, St. Louis, MO).

### Reagents

CT (List Biological Laboratories, CA, USA), CTB (List biological Laboratories), alum (Sigma-Aldrich), OVA (Sigma-Aldrich), OVA peptide_323−339_ (H-Ile-Ser-Gln-Ala-Val-His-Ala-Ala- His-Ala-Glu-Ile-Asn-Glu-Ala-Gly-Arg-OH; Sigma-Aldrich), CpG ODN (InvivoGen, CA, USA) were purchased from the indicated sources.

### ELISA

Microtiter plates (Thermo Fisher Scientific, Roskilde, Denmark) were coated with 10 μg/ml OVA for 18 h at 4°C. The Ag-coated wells were blocked with 1% bovine serum albumin (Rotech, Gunpo, Korea) for 1 h at 20°C. Each sample was diluted to the desired dilution and incubated in each well at 4°C for overnight. HRP conjugated goat anti-mouse IgG, IgG1, IgG2c, or IgA Abs (Southern biotech, Birmingham, AL) were incubated to a dilution of 1:3000 for 2 h at 20°C RT. 3,3′,5,5′-Tetramethylbenzidine (TMB, Moss Inc., Pasadena, MD) was used as substrate to visualize color change and the reaction is terminated by adding HCl. The developed colors were measured at 450 nm in an ELISA reader (Synergy HI-hybrid Reader, BioTek, Winooski, VT).

### Cell Isolation

To isolate mononuclear cells from lymphoid tissues, the cervical lymph node (CLN) or spleen was homogenized and treated with lysis buffer (Sigma-Aldrich) to remove red blood cells. CD4^+^ T cells were isolated from mononuclear cells using MagniSort^TM^ Mouse CD4 T cell Enrichment Kit (Invitrogen, CA, USA). CD11c^+^ DCs were isolated from mononuclear cells using MagniSort^TM^ Mouse CD11c Positive Selection Kit (Invitrogen).

### BMDC Generation

Bone marrow derived DCs (BMDCs) were generated by differentiating bone marrow progenitors isolated from murine femurs and tibiae in RPMI medium containing 10% heat-activated fetal bovine serum (FBS; Gibco, Paisley, Renfrewshire, UK), 10 ng/ml granulocyte-macrophage colony-stimulating factor (GM-CSF, eBioscience), and penicillin/streptomycin (Sigma-Aldrich). Culture media containing granulocyte-macrophage colony-stimulating factor (GM-CSF) were replaced with fresh medium on days 3 and 5 after bone marrow culture. On day 7 after bone marrow culture, non-adherent CD11c^+^ cells (>95%) were used for the next steps. CD11c^+^ cells at 1 × 10^6^ cells/well were stimulated with 2 μg/ml CT or 1 μM CpG ODN for 18 h. Culture supernatants were analyzed for cytokine production without further re-stimulation and cells were analyzed for expression of costimulatory molecules by flow cytometry. For western blotting assay, CD11c^+^ cells at 1 × 10^6^ cells/well were stimulated with 2 μg/ml CT for 16 h.

### Proliferation of OT-II T Cells

CD11c^+^ DCs were isolated from CLN at day 3 following intranasal CT administration. OT-II CD4^+^ T cells were isolated from spleen of OT-II mice and labeled with 0.9 μM carboxyfluorescein succinimidyl ester (CFSE; Invitrogen), a fluorescent cell-staining dye. CD11c^+^ DCs at 1×10^5^cells/well and OT-II CD4^+^ T cells at 5×10^5^ cells/well were co-cultured in the presence of 1 μM OVA peptide_323−339_ for 4 days. The cultured T cells were analyzed for CFSE fluorescent dilution by flow cytometry and the culture supernatant were used for analysis of cytokines.

### Flow Cytometry

Anti-mouse CD11c-PE, anti-mouse MHC class II I-A^b^-FITC, anti-mouse CD80-APC, anti-mouse CD86-APC, anti-mouse/rat CD40-FITC, anti-mouse CD4-FITC, anti-mouse IFN-γ-PE, and anti-mouse/rat IL17A-APC were purchased from eBioscience. Data were obtained using FACScanto or FACSAria III (BD Bioscience) and analyzed with the FlowJo software (Tree star, Ashland, OR).

### Cytokines

Murine primary CD11c^+^ DCs were isolated from the spleen or CLN of mice at day 3 following intranasal CT administration. DCs were cultured with 50 ng/ml phorbol 12-myristate 13-acetate (Sigma-Aldrich) and 1 μg/ml ionomycin (Sigma-Aldrich) in complete cell culture media for 18 h since the levels of cytokines were undetectable without re-stimulation. The culture supernatants were analyzed for cytokine production from DCs using BD^TM^ Cytometric Beads Assay Mouse inflammation kit (BD biosciences). CD4^+^ T cells were stimulated by coated 1 μg/ml anti-mouse CD3ε and 2 μg/ml anti-mouse CD28 in 96-well flat-bottomed microplates. The culture supernatant from CD4^+^ T cells or OT-II T cells were analyzed using BD^TM^ Cytometric Bead Assay Mouse Th1/Th2/Th17 CBA kit (BD biosciences) per manufacturer instructions. IL-1β was detected using mouse IL-1β ELISA MAX^TM^ Deluxe set (BioLegend, San Diego, CA) per manufacturer instruction.

### Western Blotting

SDS-PAGE and western blotting was conducted as previously described (25). Briefly, cell lysates were evaluated to determine protein levels and then loaded on SDS-PAGE gel. The protein bands were transferred to a membrane, which was subsequently incubated with primary antibodies (Abs), including anti-IRE1α Ab (3294S, Cell Signaling Technologies, MA, USA) or anti-PERK Ab (3192S, Cell Signaling Technologies), and then with secondary Abs including HRP-linked anti-rabbit IgG Ab (Cell Signaling Technologies, MA, USA), and HRP conjugated polyclonal goat anti-mouse IgG F(ab′)2 (Enzo Life Sciences, NY, USA). The substrate for enhanced chemiluminescence (ECL) was used from femtoLUCENT™ PLUS HRP Kit (G-biosciences, MO, USA). Images were obtained with ImageQuant™ LAS 4000 mini system (GE Healthcare Life Sciences, Buckinghamshire, UK) and analyzed using the Image J software (NIH, Bethesda, MD, USA).

### Statistics

Student's *t*-test or One-way ANOVA (Bonferroni's Multiple Comparison Test) were used to compare differences between the two or more than three groups. Values of *p* < 0.05 were considered significant.

## Results

### ERdj5 Is a Critical Factor in Ag-Specific Ab Production Following Mucosal Immunization With CT

**Figure 1:**
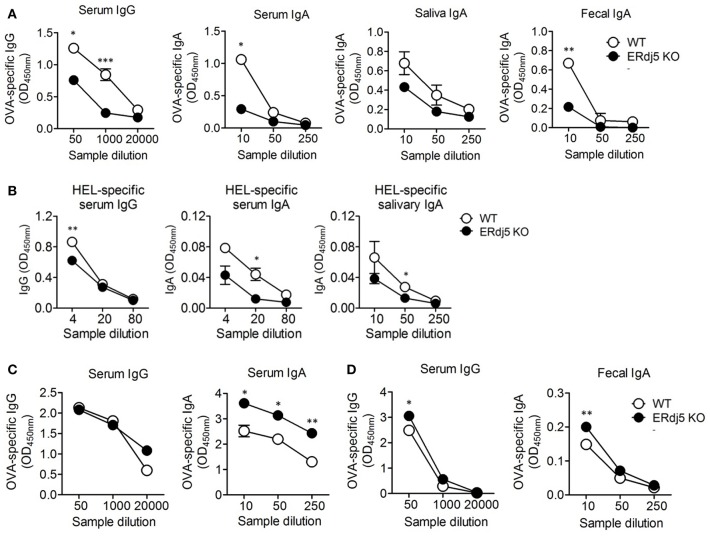
The adjuvanticity of CT significantly decreased in Erdj5 KO mice. Mice were immunized twice with 20 μg OVA plus 1 μg CT **(A)**, alum **(C)**, or CTB **(D)** at 2 weeks interval. The levels of Ag-specific Ig production were determined at 1 week following final immunization. **(A)** OVA-specific IgG or IgA in serum, saliva, and fecal extract following intranasal CT plus OVA administration (*n* = 6). **(B)** Mice were immunized twice via the intranasal route with 20 μg hen egg lysozyme (HEL) and 1 μg CT at 2 weeks interval (*n* = 3). The levels of Ag-specific Ig production were determined at 1 week following final immunization. HEL-specific IgG or IgA in serum and mucosal secretion. **(C)** OVA-specific IgG and IgA in serum following intraperitoneal 100 μg alum plus 20 μg OVA administration (*n* = 4). **(D)** OVA-specific IgG or IgA in serum and fecal extract following intranasal 1 μg CTB plus 20 μg OVA administration (*n* = 5). Student *t*-test. *p < 0.05, **p < 0.01, ***p < 0.001.

When mice were intranasally immunized with OVA alone, the level of OVA-specific IgG and IgA in the serum were identical between WT and ERdj5 KO mice (Supplementary Figure 1A). Furthermore, intranasal OVA immunization without CT could not induce mucosal IgA responses (Supplementary Figure 1B). To investigate the role of ERdj5 in the mucosal adjuvanticity of CT, wild-type (WT) and ERdj5 KO mice were intranasally administrated with OVA Ag plus CT. At 7 days following the 2nd immunization, the level of OVA-specific Ab was analyzed from serum and mucosal secretions. The level of OVA-specific IgG and IgA in the serum was significantly decreased in ERdj5 KO mice (Figure 1A). The level of OVA-specific IgA from secreted saliva and fecal extract were also decreased in ERdj5 KO mice. Even in absence of ERdj5, CT retained some adjuvanticity in the production of OVA-specific IgG in the serum when compared with OVA immunized mice (Supplementary Figure 1C). CT can be considered a protein Ag and a mucosal adjuvant. The level of CT-specific Ab was also significantly reduced in ERdj5 KO mice (Supplementary Figure 2A). When mice were intraperitoneally immunized with OVA plus CT, mucosal OVA-specific IgA responses were significantly decreased in absence of ERdj5 KO mice suggesting that reduced mucosal immune responses in the ERdj5 KO mice were independent on immunization route (Supplementary Figure 2B). To determine whether reduced CT adjuvanticity in ERdj5 KO mice can be a general event in other Ag, ERdj5 KO mice were intranasally immunized with hen egg lysozyme (HEL) plus CT. The production of HEL-specific IgG and IgA in the serum and mucosal secretion was significantly reduced in the absence of ERdj5 (Figure 1B). To investigate whether ERdj5 deficiency intrinsically affects Ab production from B cells, the levels of total IgG and IgA Abs from serum and mucosal secretion of ERdj5 KO mice were compared. The levels of total IgG and IgA in ERdj5 KO mice were similar to those in WT mice, suggesting that the B cells of ERdj5 KO mice had no defect in Ab production (Supplementary Figure 2C). Thus, the reduction of OVA-specific IgG and IgA production in ERdj5 KO mice following intranasal CT and OVA administration can be attributed to reduced adjuvanticity of CT in the absence of ERdj5. To confirm whether ERdj5 is specifically essential for CT adjuvanticity, mice were immunized intraperitoneally with OVA plus alum as an alternative vaccine adjuvant (Figure 1C). Immunization with alum could not induce OVA-specific IgA Ab secretion in the saliva or fecal extract (data not shown). In ERdj5 KO mice, the serum level of OVA-specific IgG was similar to that in WT mice, whereas the serum level of IgA was slightly higher than that in WT mice. These results suggested that ERdj5 in the ER has a critical role in Ab production induced by the mucosal adjuvanticity of whole-CT.

### ERdj5 Is Independent of the Adjuvanticity of the CTB Subunit

CT is composed of a single A subunit and five B subunits. The B subunit complex of CT (CTB) binds to the G_M1_ ganglioside receptor on the cell surface and then enters to the ER inside the cell. CTB alone without CTA can also induce immune response although the magnitude of immunity is lower than that induced by whole-CT. CTB has been developed as a candidate of safe mucosal adjuvant because it has no toxicity (26, 27). Thus, we checked whether the adjuvanticity of CTB could be associated with the presence of ERdj5. The level of OVA or CTB-specific Abs following intranasal administration of OVA plus CTB was analyzed. The levels of OVA-specific IgG in serum and IgA in fecal extract were slightly higher in ERdj5 KO mice compared to WT mice (Figure 1D). Consistent with this, the levels of CTB-specific serum IgG and fecal IgA were not decreased in ERdj5 KO mice (Supplementary Figure 2D). These results suggested that ERdj5 within ER was specifically essential for the adjuvanticity of CT via cytosolic CTA1 regardless of CTB.

### ERdj5 Deficiency Specifically Reduced the Production of Ag-Specific IgG2c Isotype

**Figure 2:**
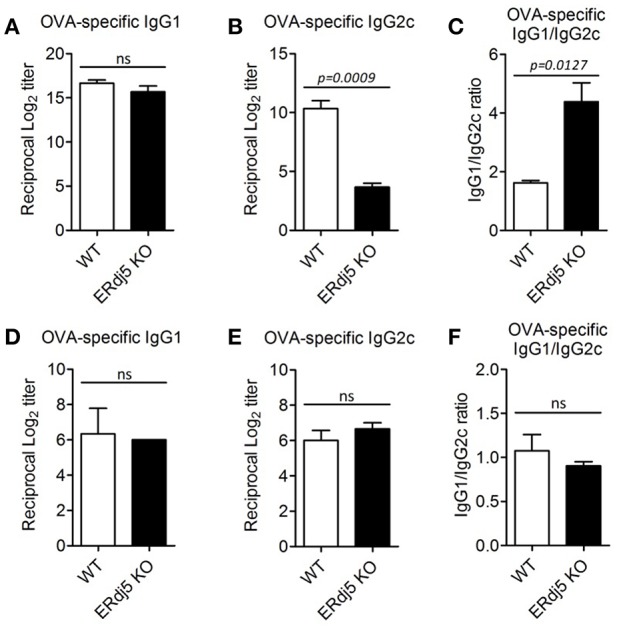
The absence of ERdj5 specifically impaired the production of IgG2c. Mice were immunized twice via the intranasal route with 20 μg OVA and 1 μg CT **(A–C)** or 1 μg CTB **(D–F)** at 2 weeks interval. The levels of Ag-specific Ig production from serum samples were determined at 1 week following final immunization. **(A,D)** The titers of OVA-specific IgG1. **(B,E)** The titers of OVA-specific IgG2c. **(C,F)** The ratio of IgG1/IgG2c. Student *t*-test.

To investigate differences in Ab production in the absence of ERdj5, isotypes of Ag-specific IgG Ab were analyzed following intranasal immunization with OVA plus CT. The titer of OVA-specific IgG1 isotype was similar between WT and ERdj5 KO mice (Figure 2A). However, the level of OVA-specific IgG2c isotype was significantly reduced in ERdj5 KO mice compared to that in WT mice, resulting in increased IgG1/IgG2c ratio (Figures 2B,C). When mice were intranasally immunized with CTB plus OVA, the levels of OVA-specific IgG1 and IgG2c were not impaired in ERdj5 KO mice compared to those in WT mice (Figures 2D–F). This result suggested that the ERdj5-dependent adjuvanticity of CT specifically induced IgG2c isotype, which might be closely related to Th1-type immune response.

### ERdj5 KO DCs Failed to Respond to CT Stimulation

**Figure 3:**
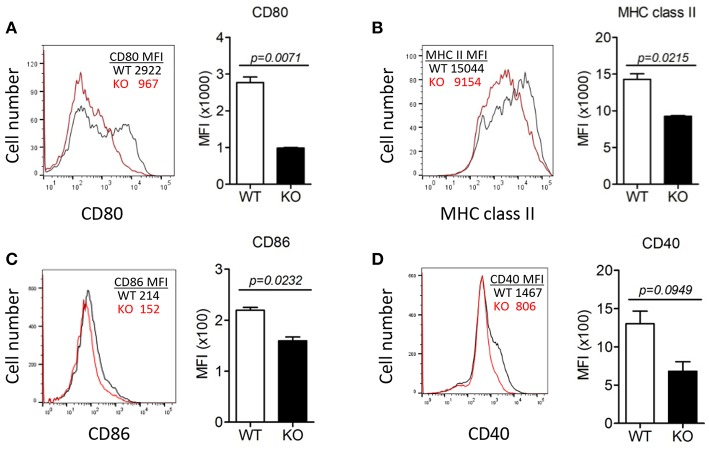
The absence of ERdj5 impaired CT-induced activation and maturation of DCs. Mice were immunized via the intranasal route with 1 μg CT. DCs were isolated from the CLN of WT (black line) or ERdj5 KO mice (red line) at 3 days following CT immunization (*n* = 4). The expression levels of co-stimulatory molecules in CD11c^+^ DCs were demonstrated as the value of mean fluorescent intensity (MFI). **(A)** Expression levels of CD80. **(B)** Expression levels of MHC class II. **(C)** Expression levels of CD86. **(D)** Expression levels of CD40. Student *t*-test.

To investigate the effect of ERdj5 deficiency on the maturation of DCs, CD11c^+^ cells were isolated from draining CLN following intranasal CT administration, and then the expression of MHC class II and costimulatory molecules was analyzed (Figure 3). The expression of CD80, MHC class II, and CD86 in the CLN DCs of ERdj5 KO mice was significantly reduced compared to that of WT mice (Figures 3A–C). The expression of CD40 in CLN DCs of ERdj5 KO mice partially decreased but the decrease was not statistically significant (Figure 3D). Without CT administration, however, the expression of costimulatory molecules on CLN DCs of ERdj5 KO mice were identical to that of WT mice (Supplementary Figure 3). In addition, the expression of costimulatory molecules in the spleen DCs of ERdj5 KO mice following CT administration was also not impaired compared to that of WT mice (Supplementary Figure 4). Specific activation of DCs from draining CLN but not from spleen implied that anatomically localized DC activation by intranasal CT ruled out the systemic effect of absorbed CT. These results suggested that DCs of ERdj5 KO mice had impaired maturation and activation capacities to initiate adaptive immune responses following CT administration.

### ERdj5 Deficiency in DCs Failed to Induce Pro-inflammatory Cytokines Following CT Stimulation

**Figure 4:**
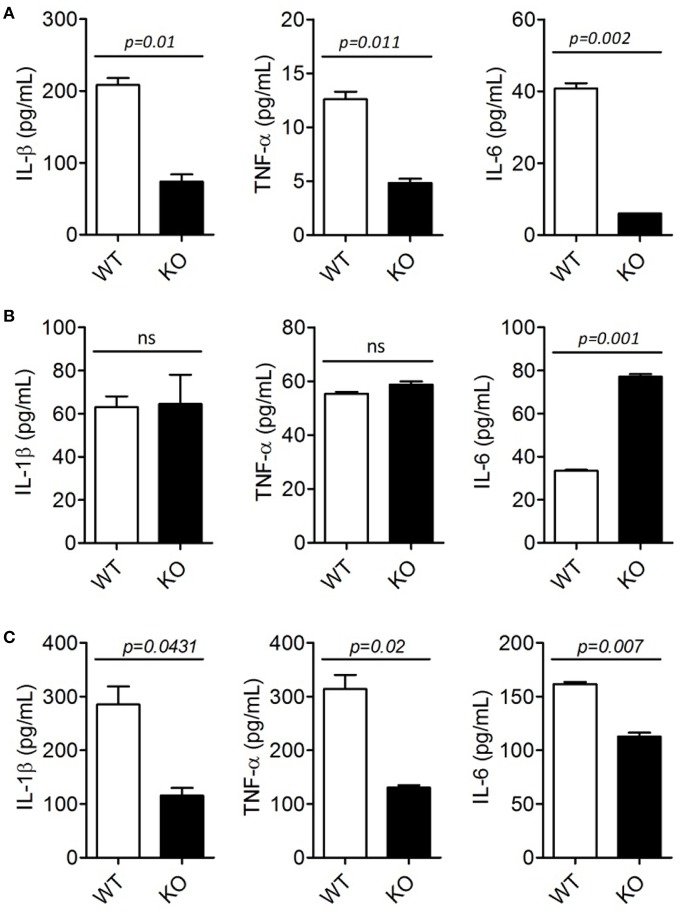
ERdj5 KO DCs failed to induce pro-inflammatory cytokines following CT treatment. **(A,B)** CD11c^+^ cells generated from BM of WT or ERdj5 KO mice were stimulated with 2 μg/ml CT (A) or 1 μM CpG **(B)** for 18 h. The levels of IL-1β, TNF-α, and IL-6 were measured from culture supernatants. **(C)** Mice were treated with 1 μg CT via the intranasal route (*n* = 4). DCs were isolated from the CLN at 3 days following CT administration. DCs were stimulated with phorbol 12-myristate 13-acetate/ionomycin and secreted cytokines, including IL-1β, TNF-α, and IL-6, were measured from culture supernatant. Student *t*-test.

To investigate whether ERdj5 deficiency affect DCs to produce pro-inflammatory cytokines following CT treatment, BMDCs generated from WT and ERdj5 KO mice were stimulated with CT or CpG. In fact, BMDCs generated from the culture of murine BM cells with GM-CSF have revealed to comprise a heterogeneous population of CD11c^+^MHCII^+^ monocyte-derived macrophages and conventional DCs as well as granulocytes (28). Therefore, we used heterogeneous CD11c^+^ populations including BMDCs to analyze the responsiveness of innate immune cells by CT. The levels of pro-inflammatory cytokines, including IL-1β, TNF-α, and IL-6, from ERdj5 KO CD11c^+^ cells significantly decreased compared to those of WT mice following CT stimulation (Figure 4A). However, after CpG treatment, the levels of IL-β and TNF-α from the ERdj5 KO CD11c^+^ cells were similar to those of WT mice (Figure 4B). IL-6 production from the ERdj5 KO CD11c^+^ cells rather increased following CpG stimulation. Without CT or CpG stimulation, the level of cytokines were undetectable (data not shown).

To investigate whether ERdj5 deficiency affects pro-inflammatory cytokine production from DCs following mucosal immunization, DCs were isolated from the CLN and spleen of mice at 3 days following intranasal CT administration. Consistent with CD11c^+^ innate immune cells generated from BM, the levels of IL-1β, TNF-α, and IL-6 from DCs isolated from the CLN of ERdj5 KO mice significantly decreased compared to those of WT mice (Figure 4C). Different from CLN, splenic DCs of ERdj5 KO mice had similar capacity to secrete pro-inflammatory cytokines to splenic DCs of WT mice, suggesting that intranasal CT administration affected the activity of DCs in local draining immune-inductive sites, but CT was not disseminated into the systemic compartment (Supplementary Figure 5). These results suggested that the absence of ERdj5 in innate immune cells such as DCs caused selective impairment of immune induction by CT stimulation.

### ERdj5 Deficiency Reduced the Generation and Differentiation of Helper T Cells

**Figure 5:**
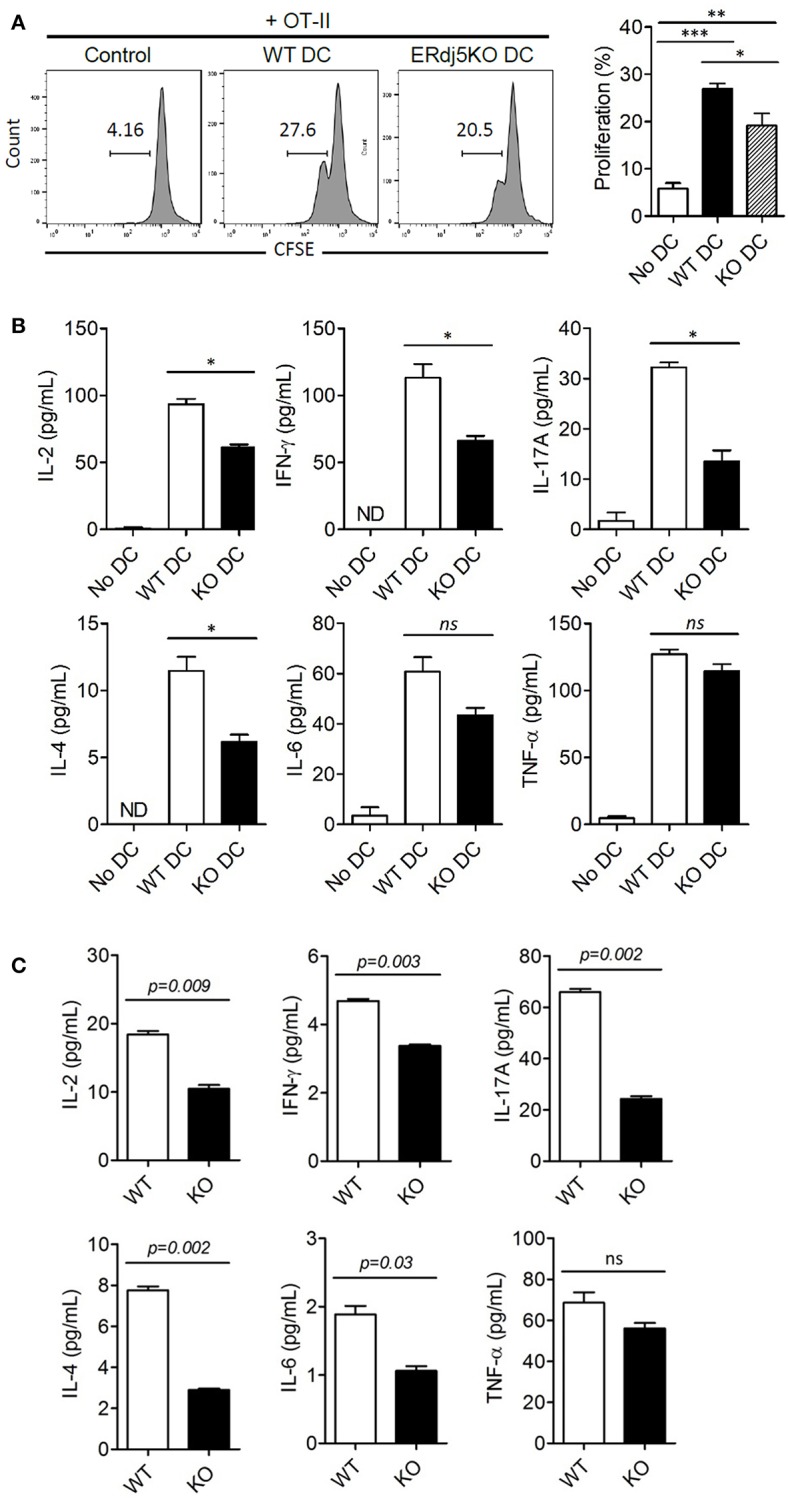
ERdj5 deficiency in DCs reduced the proliferation and differentiation of effector T cells. **(A,B)** Mice were applied with 1 μg CT via the intranasal route. At 3 days following immunization, CD 11c^+^ DCs were isolated from the CLN. CFSE-labeled OT-II CD4^+^ T cells were co-cultured with CLN DCs for 4 days. The proliferation of OT-II CD4^+^ T cells were determined by CFSE dilution. One-way ANOVA (Bonferroni's Multiple Comparison Test). *p < 0.05, **p < 0.01, ***p < 0.001. **(B)** The cytokine production, including IL-2, IFN-γ, IL-17A, IL-4, IL-6, and TNF-α were determined from culture supernatants of OT-II and CLN DCs. ND, not detected. One-way ANOVA (Bonferroni's Multiple Comparison Test). ns, not significant. *p < 0.05. **(C)** Mice were immunized twice with 1 μg CT and 20 μg OVA via the intranasal route at 2 weeks intervals (*n* = 4). At 3 days following final immunization, CD4^+^ T cells were isolated from the spleen of immunized mice. T cells were stimulated with plate-bound anti-CD3/anti-CD28 for 24 h and the productions of cytokines, including IL-2, IFN-γ, IL-17A, IL-4, IL-6, and TNF-α, from culture supernatant were determined. Student *t*-test.

Impairment of innate immune responses, especially in DCs in the absence of ERdj5, implied reduced adaptive immune responses. To investigate whether DCs from ERdj5 KO mice fail to prime CD4^+^ T cells, DCs were isolated from the CLN at 3 days following CT administration. The proliferation of OT-II CD4^+^ T cells co-cultured with DCs of ERdj5 KO mice significantly decreased (Figure 5A). To investigate the differentiation of CD4^+^ T cells into helper T cells, the cytokine profiles from culture supernatants of OT-II CD4^+^ T cells activated by CLN DCs were determined. The levels of IL-2, IFN-γ, IL-17A, and IL-4 from OT-II CD4^+^ T cells activated by ERdj5 KO DCs were significantly decreased compared to those of T cells activated by WT DCs (Figure 5B). These results suggested that ERdj5 KO DCs had reduced activation status in response to CT (Figures 3, 4) and resulted in reduced priming and differentiation of CD4^+^ T cells. To confirm the reduced responses of CD4^+^ T cells in ERdj5 KO mice, CD4^+^ T cells were isolated from the spleen following two times of intranasal immunization with CT plus OVA and analyzed for cytokine signatures of helper T cell subsets. The levels of IL-2, IFN-γ, IL-17A, IL-4, and IL-6 secreted from CD4^+^ T cells from ERdj5 KO mice significantly decreased compared to those of WT mice (Figure 5C). The level of TNF-α from CD4^+^ T cells from the CLN of ERdj5 KO mice was not significantly different from that of WT mice. Intranasal OVA immunization without CT could not induce considerable cytokines from CD4^+^ T cells (data not shown). Collectively, these results suggested that ERdj5 deficiency reduced the generation of most helper T cell subsets, including Th1 (producing IL-2 and IFN-γ), Th2 (producing IL-4 and IL-6), and Th17 (producing IL-17A) via reduced activation of ERdj5 KO DCs in response to CT.

### ERdj5 Deficiency Enhanced ER Stress by CT in DCs

**Figure 6:**
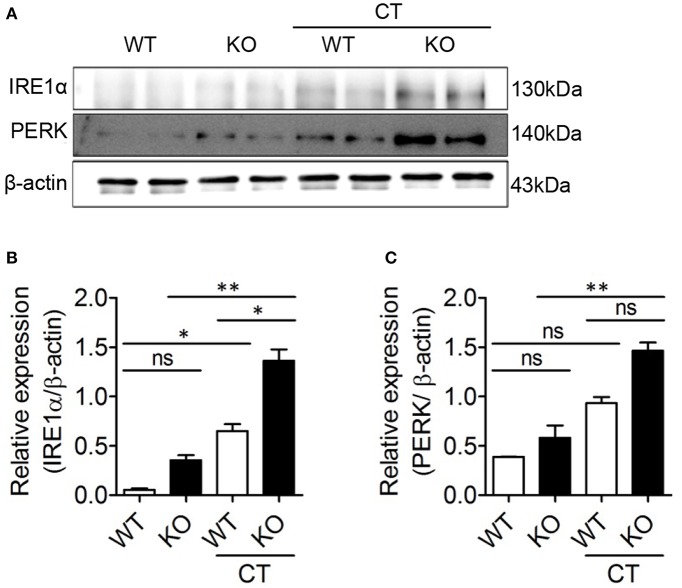
CT enhanced ER stress in the ERdj5 KO CD11c^+^ cells. CD11c^+^ cells generated from BM of WT or ERdj5 KO mice were treated with 2 μg CT for 16 h. **(A)** The expression levels of IRE1α and PERK proteins were analyzed by western blotting. Relative band intensities of IRE1α **(B)** and PERK **(C)** compared to that of β-actin were summarized. One-way ANOVA. *p < 0.05, **p < 0.01.

ERdj5 deficiency failed to induce sufficient adjuvanticity of CT. However, the production of IgG1 Abs, CD4^+^ T cell priming, and several immune responses were reduced but retained in ERdj5 KO mice. One possibility for retained adjuvanticity of CT partially can be ascribable to an incomplete block of CTA1 translocation upon ERdj5 knockdown (24). In addition, CTB has been revealed to upregulate ER stress molecules (29). We investigate whether these events in the absence of ERdj5 can induce ER stress in DCs. CD11c^+^ cells generated from BM of WT or ERdj5 KO mice were treated with CT for 16 h and then the expression levels of IRE 1α and PERK, representative ER stress parameters, were evaluated (Figure 6A). At a steady condition, the expression of IRE 1α and PERK in the ERdj5 KO CD11^+^ cells was similar to that of WT mice. Upon CT stimulation, the expression of IRE 1α and PERK significantly increased in the ERdj5 KO cells compared to that of WT mice, suggesting enhanced ER stress (Figures 6B,C). These might contribute to the retained adjuvanticity of CT in ERdj5 KO mice in the absence of translocated CTA1-induced inflammatory signals. Consistent with this, intranasal immunization with OVA plus CTB induced slightly higher level of serum IgG and fecal IgA in the ERdj5 KO mice (Figure 1D).

## Discussion

**Figure 7:**
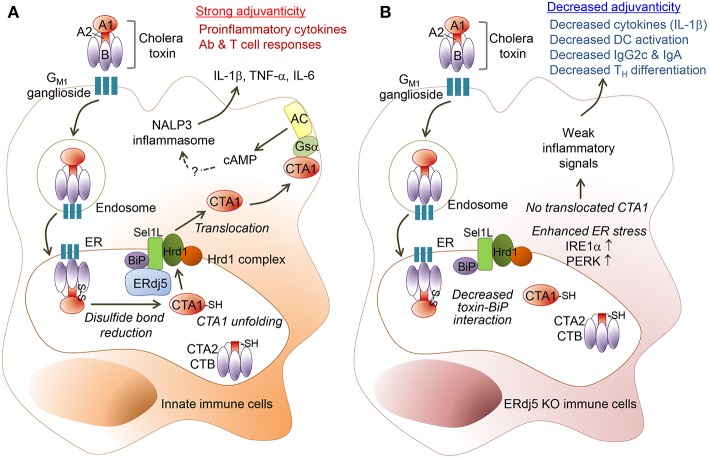
ERdj5 is a critical factor for the adjuvant effect of CT. **(A)** CT contains a single A and five B subunits. CT can bind with G_M1_ ganglioside on the cell surface via the B subunit of CT (CTB) and then it can be internalized by an endosome and proceed into ER. Within the ER, CTA1 chain is released by the reduction of a disulfide bridge from CTB-CTA2 complex. ERdj5, by binding to Sel1L, trigger BiP-CTA1 interaction to the Hrd1 complex on ER membrane to retro-translocate CTA1 into the cytosol. Cytosolic CTA1 can bind to Gsα and finally activate adenylate cyclase (AC) to make intracellular cAMP. Elevated intracellular cAMP induces pro-inflammatory cytokines, such as IL-1β, TNF-α, and IL-6. Finally, CT stimulates innate immune cells as a strong mucosal adjuvant to induce adaptive immune responses, including Ab production and T cell responses in the systemic and mucosal compartment. **(B)** In absence of ERdj5 molecule, CTA1 cannot retro-translocate to the cytosol and induce inflammatory signals. Instead, the expression of IRE1α and PERK increased in ERdj5 KO innate immune cells, suggesting enhanced ER stress. Collectively, ERdj5 KO innate immune cells showed decreased levels of pro-inflammatory cytokines such as IL-1β and costimulatory molecules, leading to decreased IgG2c and IgA levels as well as T_H_ differentiation.

CT enters from the host cell surface into the ER, where the CTA1 subunit dissociates from the CT complex and then is translocated into the cytosol to induce toxicity in the enterocytes or inflammatory cascades in immune cells (24). The ER-resident factor ERdj5, by binding to Sel1L, facilitates the interaction of the CTA1 subunit to Hrd1-associated retro-translocation machinery. Therefore, ERdj5-knockdown can be expected to reduce Hrd1–CTA1 interaction and ultimately reduce CTA1 retro-translocation. However, it has not been fully elucidated whether ERdj5 deficiency in the enterocytes can reduce host cellular toxicity. In immune cells, CT induces inflammatory cytokines, which leads to adjuvanticity, and induces strong innate and adaptive immunity against the Ag administered together with CT. Studies regarding the adjuvanticity of CT in the absence of ERdj5 has not been revealed. In the current study, we investigated whether ERdj5 is a crucial factor for the adjuvanticity of CT to induce systemic and mucosal immune responses using ERdj5 KO mice. In ERdj5 KO mice, the level of Ag-specific IgG in the serum as well as IgA in the mucosal secretion significantly decreased. Therefore, ERdj5 was proved to play a critical role in CTA1 induced adjuvanticity (Figure 7).

In the absence of ERdj5, the level of Ag-specific IgG2c isotype was significantly reduced following intranasal administration of CT plus Ag, although the level of IgG1 isotype was not changed. When CTB and Ag were intranasally administered, the level of IgG2c in ERdj5 KO mice was similar to that in WT mice. These results suggested that ERdj5-mediated CTA1 retro-translocation might be crucial in the induction of Th1 responses supporting IgG2c production. In fact, IFN-γ-producing Th1 cells also decreased in the spleen of ERdj5 KO mice. However, the production of IL-12p70 and IFN-γ from DCs isolated from the CLN of ERdj5 KO mice following intranasal administration of CT were comparable to that of WT mice (data not shown). Instead, IL-1β and IL-6 production from DCs of ERdj5 KO mice significantly decreased, leading to decreased Th17 differentiation. Consistent with this, IL-1R KO mice showed decreased Th17 cells, leading to reduced IgG2a or IgG2c against *Bordetella pertussis* (30). In the spleen of ERdj5 KO mice, the population of IL-17A-secreting Th17 decreased more dramatically than that of Th1. Therefore, decreased IgG2c production in the absence of ERdj5 may be attributed to decreased Th17 generation caused by decreased IL-1β.

Similar to decreased production of pro-inflammatory cytokines from CLN DCs of ERdj5 KO mice, the expression of MHC class II and costimulatory molecules, such as CD80, CD86, and CD40, also decreased in the CLN DCs of ERdj5 KO mice. When the DCs of ERdj5 KO mice were stimulated with CpG, a TLR9 ligand, they produced similar levels of pro-inflammatory cytokines to those produced by the DCs of WT mice, suggesting that ERdj5 specifically mediated the activation of DCs induced by CT stimulus.

Our results clearly showed that ERdj5 was a critical host factor for the strong adjuvanticity of CT. However, there are several points that are still questionable. CT had significantly decreased but retained its adjuvanticity, especially in serum IgG1 production in ERdj5 KO mice. It still needs to be investigated whether ER-retained CT might have some activity to induce immune responses without increased cytosolic cAMP machinery by cytosolic CTA1. In fact, the CTB subunit can also exert adjuvanticity even though it has milder activity than the CTA1 domain. Even CTB itself can induce NLRP3 inflammasome to produce IL-1β via the caspase-1 pathway (31). In our result, the CTB subunit can induce Ag-specific Ab production, but ERdj5 is not required for the activity of CTB.

The underlying immunostimulatory mechanisms of various adjuvants have been investigated to develop human vaccines. A squalene-based oil-in-water emulsion, AS03, can induce immunostimulation via activation of IRE1α, an ER stress sensor (32). Glucocorticoids can contribute to immunostimulation on macrophages, which may be correlated to ER stress via the glucocorticoid receptor (33). CTB can upregulate ER stress proteins (29). Consistent with this, our result also confirmed that CT enhanced IRE 1α expression in the CD11c^+^ cells generated from WT BM. In the ERdj5 KO CD11c^+^ cells, IRE 1α and PERK increased more significantly, suggesting enhanced ER stress. Therefore, remaining immunostimulatory effect of CT in ERdj5 KO mice could be expected during enhanced ER stress as well as incomplete blockade of CTA1 retrotranslocation (24).

One of the critical steps to develop safe and effective vaccines is choosing a suitable immunological adjuvant to induce humoral and/or cell-mediated immune responses to vaccine Ags (34). Our results showed that ERdj5 is a critical factor for the adjuvanticity of CT via mediation of CTA1 retro-translocation. *Vibrio cholera* utilizes the host factor ERdj5 to exert its pathogenicity. In turn, we utilized bacterial toxin to acquire sufficient host protective immunity. ERdj5 is important for the strong mucosal adjuvanticity of CT in innate immune cells. These mechanistic studies for CT provided valuable information for the development of novel, safe, and effective mucosal adjuvants.

## Ethics Statement

All experiments were approved by the Institutional Animal Care and Use committee of Ajou University (IACUC approval number 2016-0034).

## Author Contributions

M-SK, H-JK, and S-YC conceived and designed experiments. M-SK, E-JY, Y-IK, and S-RK performed experiments and analysis. TI, SK, and Y-SJ provided critical materials. M-SK, S-RK, H-JK, and S-YC wrote the manuscript and provided creative input.

### Conflict of Interest Statement

The authors declare that the research was conducted in the absence of any commercial or financial relationships that could be construed as a potential conflict of interest.
