# Supplementary material for: ERdj5 in Innate Immune Cells Is a Crucial Factor for the Mucosal Adjuvanticity of Cholera Toxin
Source: Front Immunol. 2019 Jun 4;10:1249. doi: 10.3389/fimmu.2019.01249 (PMC6593289; doi:10.3389/fimmu.2019.01249)
Supplement: Supplementary file 1 [file Presentation_1.pptx]

## Slide 1
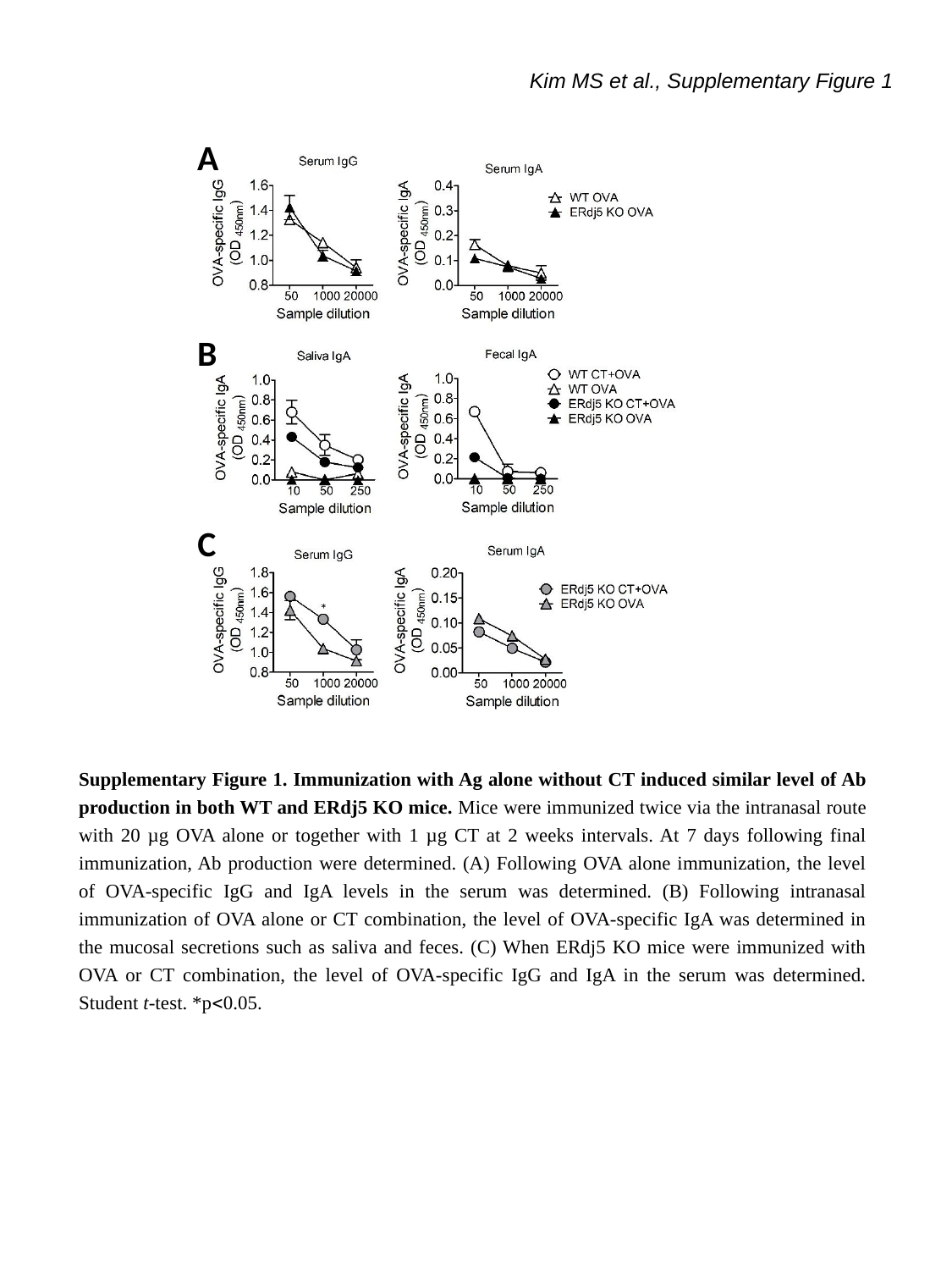

Kim MS et al., Supplementary Figure 1
A
B
C
Supplementary Figure 1. Immunization with Ag alone without CT induced similar level of Ab production in both WT and ERdj5 KO mice. Mice were immunized twice via the intranasal route with 20 µg OVA alone or together with 1 µg CT at 2 weeks intervals. At 7 days following final immunization, Ab production were determined. (A) Following OVA alone immunization, the level of OVA-specific IgG and IgA levels in the serum was determined. (B) Following intranasal immunization of OVA alone or CT combination, the level of OVA-specific IgA was determined in the mucosal secretions such as saliva and feces. (C) When ERdj5 KO mice were immunized with OVA or CT combination, the level of OVA-specific IgG and IgA in the serum was determined. Student t-test. *p0.05.

## Slide 2
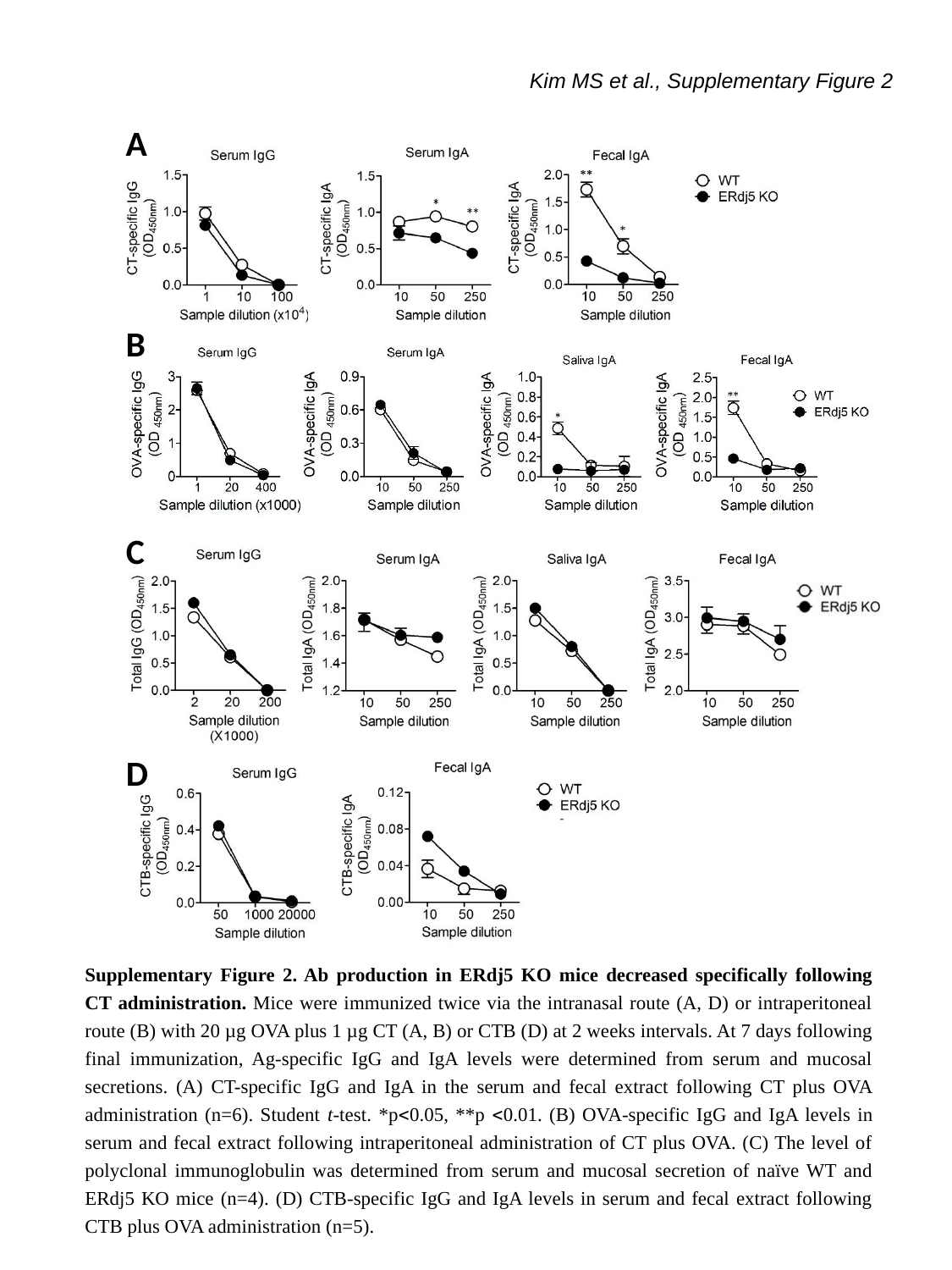

Kim MS et al., Supplementary Figure 2
A
B
C
D
Supplementary Figure 2. Ab production in ERdj5 KO mice decreased specifically following CT administration. Mice were immunized twice via the intranasal route (A, D) or intraperitoneal route (B) with 20 µg OVA plus 1 µg CT (A, B) or CTB (D) at 2 weeks intervals. At 7 days following final immunization, Ag-specific IgG and IgA levels were determined from serum and mucosal secretions. (A) CT-specific IgG and IgA in the serum and fecal extract following CT plus OVA administration (n=6). Student t-test. *p0.05, **p 0.01. (B) OVA-specific IgG and IgA levels in serum and fecal extract following intraperitoneal administration of CT plus OVA. (C) The level of polyclonal immunoglobulin was determined from serum and mucosal secretion of naïve WT and ERdj5 KO mice (n=4). (D) CTB-specific IgG and IgA levels in serum and fecal extract following CTB plus OVA administration (n=5).

## Slide 3
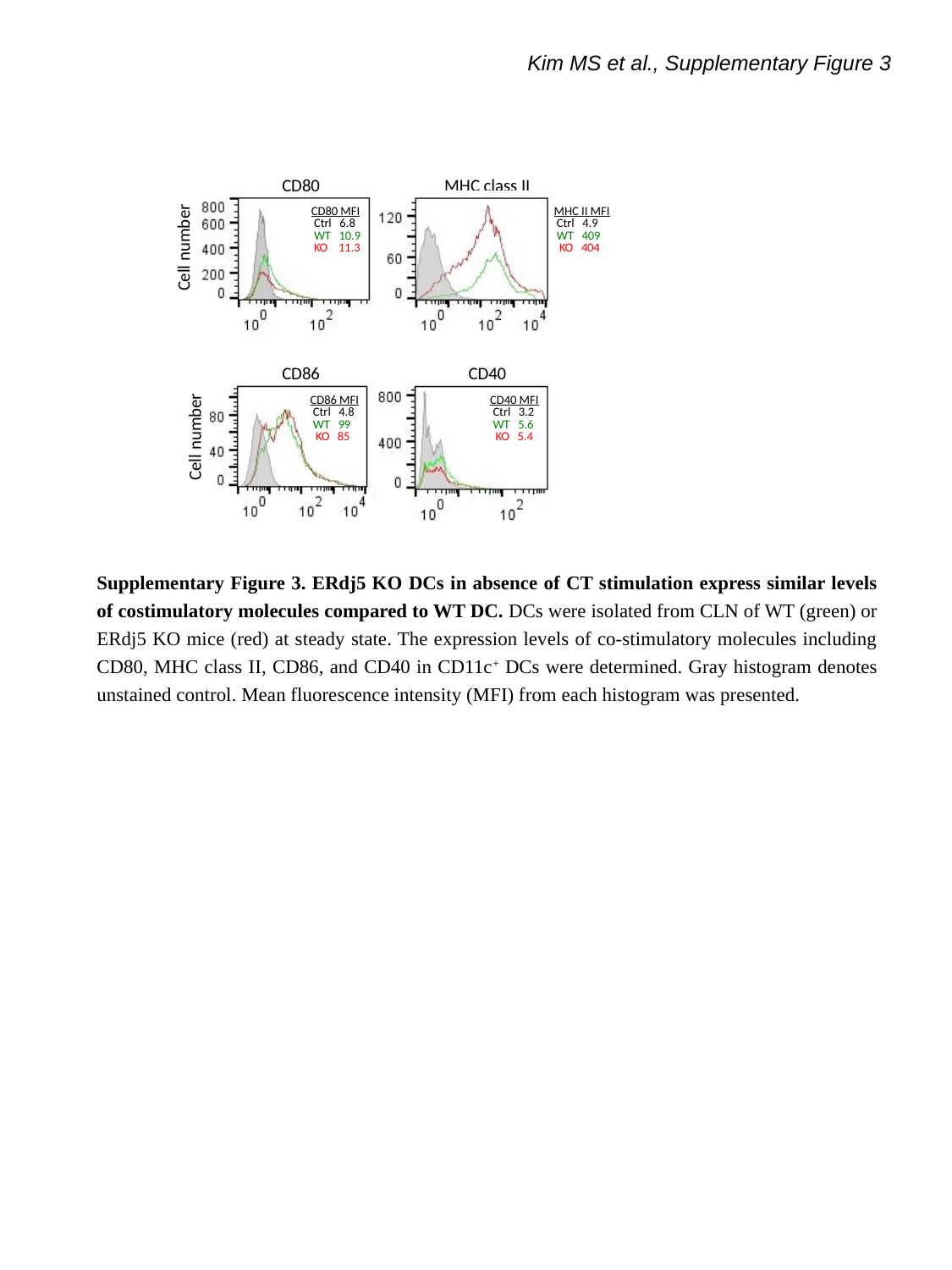

Kim MS et al., Supplementary Figure 3
CD80
MHC class II
CD80 MFI
 Ctrl 6.8
 WT 10.9
 KO 11.3
MHC II MFI
 Ctrl 4.9
 WT 409
 KO 404
Cell number
CD86
CD40
CD86 MFI
 Ctrl 4.8
 WT 99
 KO 85
CD40 MFI
 Ctrl 3.2
 WT 5.6
 KO 5.4
Cell number
Supplementary Figure 3. ERdj5 KO DCs in absence of CT stimulation express similar levels of costimulatory molecules compared to WT DC. DCs were isolated from CLN of WT (green) or ERdj5 KO mice (red) at steady state. The expression levels of co-stimulatory molecules including CD80, MHC class II, CD86, and CD40 in CD11c+ DCs were determined. Gray histogram denotes unstained control. Mean fluorescence intensity (MFI) from each histogram was presented.

## Slide 4
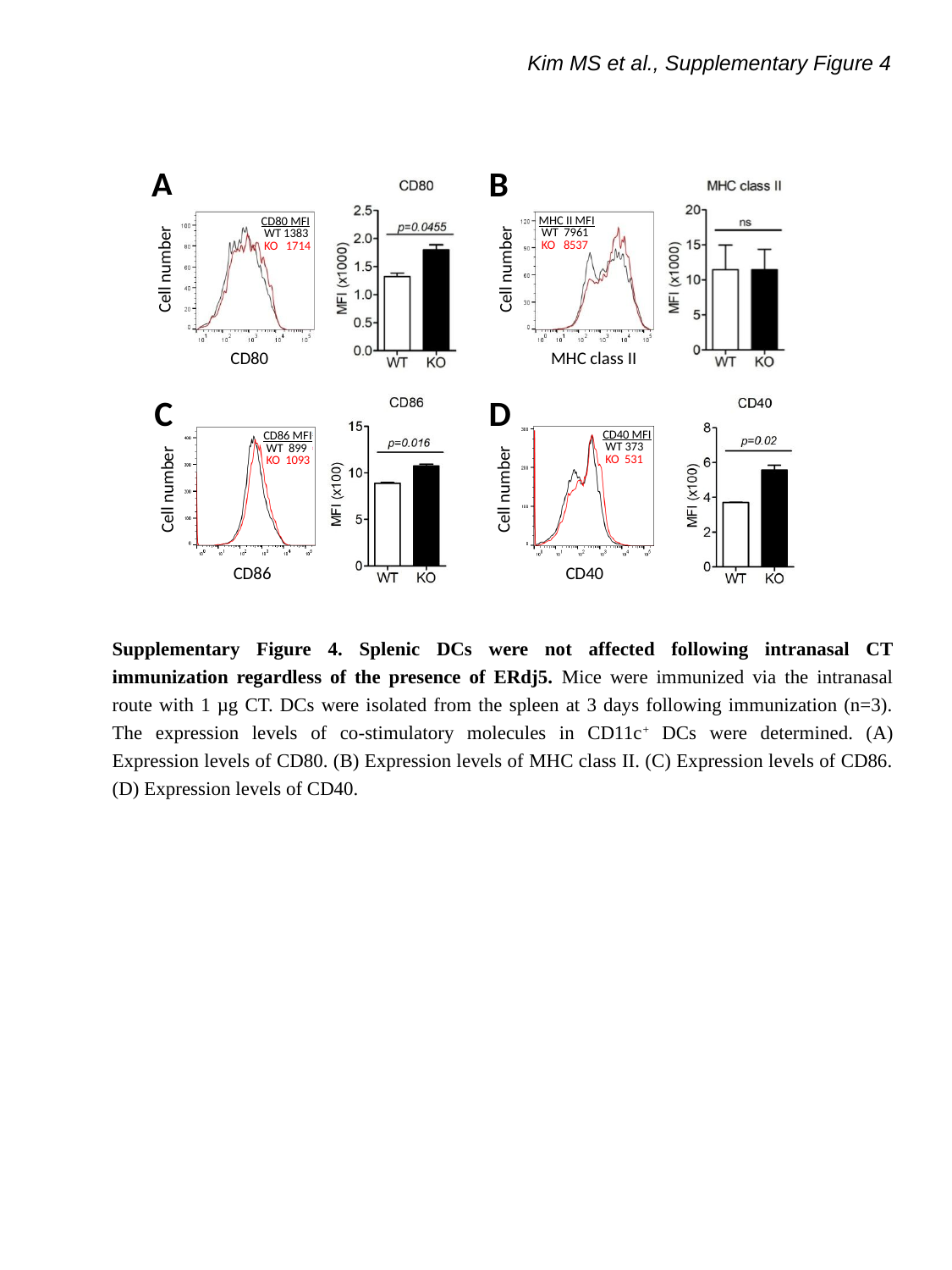

Kim MS et al., Supplementary Figure 4
A
B
Cell number
MHC class II
MHC II MFI
 WT 7961
 KO 8537
CD80 MFI
 WT 1383
 KO 1714
Cell number
CD80
C
Cell number
CD86
D
Cell number
CD40
CD40 MFI
 WT 373
 KO 531
CD86 MFI
 WT 899
 KO 1093
Supplementary Figure 4. Splenic DCs were not affected following intranasal CT immunization regardless of the presence of ERdj5. Mice were immunized via the intranasal route with 1 µg CT. DCs were isolated from the spleen at 3 days following immunization (n=3). The expression levels of co-stimulatory molecules in CD11c+ DCs were determined. (A) Expression levels of CD80. (B) Expression levels of MHC class II. (C) Expression levels of CD86. (D) Expression levels of CD40.

## Slide 5
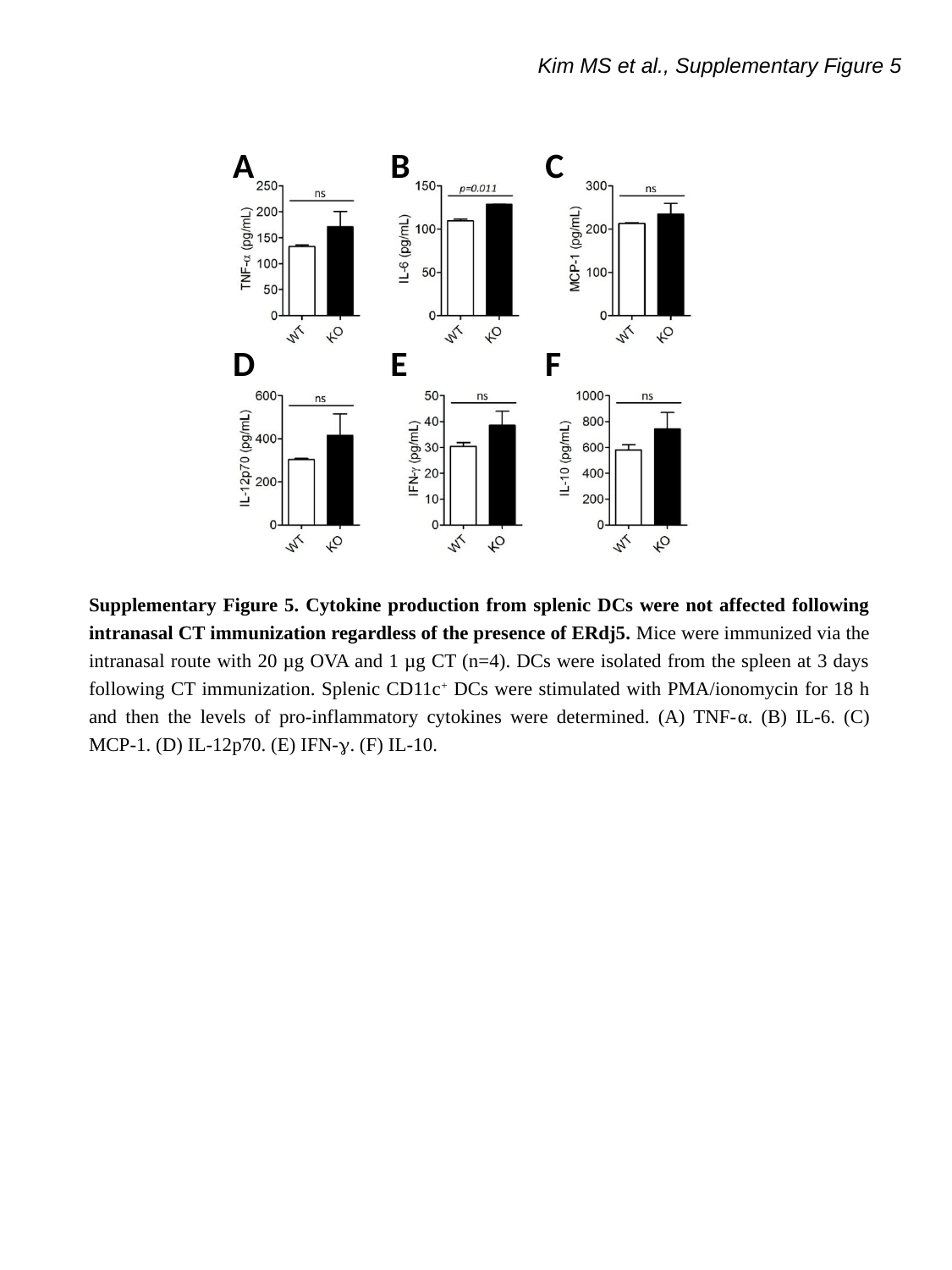

Kim MS et al., Supplementary Figure 5
A
B
C
D
E
F
Supplementary Figure 5. Cytokine production from splenic DCs were not affected following intranasal CT immunization regardless of the presence of ERdj5. Mice were immunized via the intranasal route with 20 µg OVA and 1 µg CT (n=4). DCs were isolated from the spleen at 3 days following CT immunization. Splenic CD11c+ DCs were stimulated with PMA/ionomycin for 18 h and then the levels of pro-inflammatory cytokines were determined. (A) TNF-α. (B) IL-6. (C) MCP-1. (D) IL-12p70. (E) IFN-γ. (F) IL-10.
